# Basic and clinical immunology – 3028. Interaction of DNA methylation and genetic variants of IL13 is associated with FEV1/FVC and BHR

**DOI:** 10.1186/1939-4551-6-S1-P203

**Published:** 2013-04-23

**Authors:** Veeresh K Patil, John Holloway, H Zhang, S Ewart, SH Arshad, W Karmaus

**Affiliations:** 1 David Hide Asthma & Allergy Research Centre, Isle Of Wight, UK; 2 Faculty of Medicine, University of Southampton, UK; 3 University of South Carolina, USA; 4 Michigan State University, USA

## Background

*IL13* genetic polymorphisms together with environmental exposures such as tobacco smoke exposure have been associated with asthma and lung function. However, both genetic and epidemiological studies fail to explain the variability in susceptibility and phenotypes. The epigenome, particularly DNA methylation (DNA-M), represents a site of molecular interaction between the environment and the genome, regulates gene expression and plausibly plays an important role in determining phenotype.

## Aims

To see if methylation is genotype dependent in *IL13* and to assess the effect of the interaction between *IL13* SNPs and DNA methylation on FEV1/FVC and BHR.

## Methods

Subjects from the 1989 Isle of Wight birth cohort (n=1456) were followed up at 1, 2, 4, 10 and 18 years. At 18 years 839/1313 had spirometry, 585/1313 had bronchial challenge. Illumina Human450K methylation arrays were used to assess DNA methylation (DNA-M) levels at >484,000 CpG sites in 245 female participants. DNA-M in three *IL13* promoter region CpG sites; cg13566430, cg14523284 and cg06584121 were explored among genotypes of three *IL13* SNP`s that have been shown to be associated with asthma related lung function measurements. Outcomes are FEV1/FVC from spirometry and DRS (Dose Response Curve): gradient of the linear regression line for FEV_1_ drop from baseline with each successive incremental dose of methacholine administered. Kruskall-Wallis tests were used to assess genotype dependent methylation and linear regression and linear mixed models were used to investigate interactions between SNPs and DNA-M.

## Results

Methylation levels were significantly different (p<0.001) for cg13566430 across the genotypes of all three SNPs. An interaction effect for rs1800925*cg13566430 was seen for both FEV1/FVC (p=0.013) and DRS (p= 0.036). For DRS; interactions of rs20541 with cg13566430 (p=0.030) and cg0658412 (p=0.036), rs2243204*cg13566430 (p=0.005), rs2243204*cg0658412 (p=0.012) and rs2243204*cg14523284 (p=0.017) were significant.

## Conclusions

Genotype-dependent DNA methylation was seen at cg13566430. Interaction between genotype and DNA methylation was significantly associated with obstructive (FEV1/FVC) and reactive (DRS) aspects of asthma in adolescent females. DNA methylation is likely to modify the effect size of the impact of genetic variants that play a role in the development of complex traits like asthma.

